# Evaluating the Efficacy of a Pre-Established Lipid-Lowering Algorithm in Managing Hypercholesterolemia in Patients at Very High Cardiovascular Risk

**DOI:** 10.3390/jpm14101044

**Published:** 2024-10-09

**Authors:** Jean Philippe Henry, Laurence Gabriel, Maria-Luiza Luchian, Julien Higny, Martin Benoit, Olivier Xhaët, Dominique Blommaert, Alin-Mihail Telbis, Benoit Robaye, Antoine Guedes, Fabian Demeure

**Affiliations:** Department of Cardiology, Université Catholique de Louvain, CHU UCL Namur, 5530 Yvoir, Belgium; laurence.gabriel@chuuclnamur.uclouvain.be (L.G.); marialuiza.luchian@yahoo.com (M.-L.L.); julien.higny@chuuclnamur.uclouvain.be (J.H.); martin.benoit@chuuclnamur.uclouvain.be (M.B.); olivier.xhaet@chuuclnamur.uclouvain.be (O.X.); dominique.blommaert@chuuclnamur.uclouvain.be (D.B.); mihail_telbis@yahoo.co.uk (A.-M.T.); benoit.robaye@chuuclnamur.uclouvain.be (B.R.); antoine.guedes@chuuclnamur.uclouvain.be (A.G.); fabian.demeure@chuuclnamur.uclouvain.be (F.D.)

**Keywords:** hypercholesterolemia, lipid-lowering treatment, cardiovascular risk, combination therapy

## Abstract

Background: Recent data from European studies (EUROASPIRE V, DA VINCI, SANTORINI) indicate that achieving the LDL cholesterol (LDL-C) target in patients at very high cardiovascular risk is uncommon. Additionally, using a combination therapy involving statins and ezetimibe remains infrequent. Methods: A single-center assessment of a pre-defined lipid lowering treatment algorithm’s effectiveness at achieving the LDL-C target in patients at very high cardiovascular risk one month and one year after hospitalization. Results: 81 patients were included, all in secondary prevention. The average age of the patient was 66.9 years, and the main cardiovascular risk factors included hypertension, diabetes mellitus, and smoking history. Following the predefined lipid-lowering algorithm specific to our study, which involves initiating high-intensity statin therapy or a combination of statin and ezetimibe depending on initial LDL-C levels and patient history; 30 (37%) patients initiated high-intensity statin therapy (Atorvastatin (40 mg, 80 mg) or Rosuvastatin (20 mg, 40 mg)), while 51 (63%) started combination therapy with high-intensity statin and ezetimibe 10 mg. After one year, 57 (70.4%) remained adherent to their initial treatment, achieving a mean LDL-C of 49.5 ± 16.9 mg/dL, with 36 (63.2%) of them reaching the LDL-C target of <55 mg/dL. A total of 13 patients discontinued treatment, and 9 were lost to follow-up, withdrew from the study, or died. Conclusion: Initiating dual statin and ezetimibe therapy or high-intensity statin therapy early, based on the expected treatment efficacy, holds the potential to more rapidly and effectively achieve LDL-C targets in a larger proportion of very high-risk cardiovascular patients.

## 1. Introduction

In 2019, the European Society of Cardiology (ESC) and the European Society of Atherosclerosis (EAS) introduced updated guidelines, lowering the therapeutic targets for lipid-lowering therapy [1]. Notably, targets for very high cardiovascular risk patients were reduced from 70 mg/dL (1.8 mmol/L) to 55 mg/dL (1.4 mmol/L), with the additional goal of achieving a 50% reduction from the initial value. These new targets [1] present challenges for implementation in clinical practice. The primary cardiovascular risk factors include hypertension, hyperlipidemia, diabetes, smoking, obesity, physical inactivity, family history of cardiovascular disease [2].

Very high cardiovascular risk is defined by specific characteristics, including [1]:

- Documented atherosclerotic cardiovascular disease (ASCVD), either clinically or on imaging. ASCVD includes acute coronary syndrome (ACS) (myocardial infarction or unstable angina), coronary revascularization (angioplasty, bypass surgery or other arterial revascularization), stroke, transient ischemic attack and peripheral arterial disease. Imaging-documented ASCVD includes findings known to be predictive of clinical events, such as significant atheromatous plaque on coronary angiography, CT scan or carotid ultrasound;
- Diabetes mellitus with target organ involvement (microalbuminuria, retinopathy or neuropathy) or ≥3 major cardiovascular risk factors or long-standing type I diabetes (>20 years);
- Severe chronic renal failure (eGFR < 30 mL/min/1.73 m^2^);
- Ten-year cardiovascular risk ≥ 10% according to the SCORE table [3];
- Familial hypercholesterolemia with documented atherosclerotic cardiovascular disease or at least one major cardiovascular risk factor.

Statins have long been the only therapeutic option for lowering LDL cholesterol (LDL-C) [4,5,6]. What is well demonstrated by recent research [7,8,9] is that the cardiovascular benefit does not depend on the specific molecule used, but rather on the shared mechanism by which these treatments lower LDL cholesterol. This mechanism involves upregulating LDL receptors on the surface of hepatocytes, thereby increasing LDL clearance from the bloodstream. This principle has been consistently supported by various studies, such as the consensus statement from the European Atherosclerosis Society Consensus Panel, which highlighted evidence from genetic, epidemiologic, and clinical studies demonstrating the causal role of LDL-C in atherosclerotic cardiovascular disease [7]. Furthermore, a meta-analysis of 170,000 participants across 26 randomized trials confirmed that intensive lowering of LDL-C, irrespective of the specific agent used, effectively reduces cardiovascular risk [9]. A more recent meta-analysis reinforced these findings by showing that intensive LDL-C lowering is both effective and safe even in older patients, who represent a high-risk group [8].

Current guidelines from the European Society of Cardiology (ESC) [1] recommend a three-stage approach to dyslipidemia management. First, initiating high-intensity statin therapy, followed by adding ezetimibe [10] if the target is not achieved and, third, if necessary, introducing a PCSK9 inhibitor [11]. Common side effects of statin therapy include myalgia, liver enzyme abnormalities, and, rarely, rhabdomyolysis [4,5]. Ezetimibe is generally well tolerated but can cause gastrointestinal discomfort [11].

While real-world data from European registries [12,13,14] have consistently revealed that LDL-C targets for high and very high cardiovascular risk patients are frequently unattained and that the utilization of dual therapy with statins and ezetimibe remains uncommon, it is important to highlight that current practice often fails to prioritize lipid profile monitoring and essential treatment adjustments [12,13,14].

### 1.1. DA VINCI Study [12]

The observational DA VINCI study [12], conducted in 18 European countries between June 2017 and November 2018, investigated lipid-lowering treatment prescription in primary and secondary prevention. In the DA VINCI study [12], out of a total of 5888 patients at very high cardiovascular risk, 2775 (47.1%) were in primary prevention and 3113 (52.9%) were in secondary prevention. The study [12] revealed that high-intensity statin monotherapy was prescribed to only 20% of very high cardiovascular risk patients in primary prevention and 37.5% in secondary prevention. In contrast, only 9% of very high-risk patients (both primary and secondary prevention) received combination therapy comprising a moderate- or high-intensity statin and ezetimibe.

Furthermore, even with all treatment modalities combined, only 18% of very high-risk patients reached their LDL-C targets. High-intensity statins achieved targets in 22% of patients, and combination therapy achieved targets in only 20%.

### 1.2. EUROASPIRE V Study [13]

The EUROASPIRE V study [13], published in 2019, aimed to assess the implementation of European recommendations in the management of dyslipidemia in coronary artery disease patients (secondary prevention).

This study [13] found that 49.9% of patients were treated with high-intensity statins and only 2.7% received combination therapy with statin and ezetimibe. After 4 to 12 weeks of treatment, less than 20% of patients achieved the previous targets applied during the study period (according to 2016 guidelines [15]), i.e., LDL-C < 70 mg/dL. Achievement of this target was only 36.6% in patients on high-intensity statin therapy.

Notably, treatment intensification strategies during follow-up were often ineffective, with treatments more commonly being reduced in intensity and rarely escalated, even for patients not reaching their therapeutic goals.

### 1.3. SANTORINI Study [14]

The SANTORINI study [14], a multicenter prospective study designed to evaluate, under real-life conditions, the efficacy of lipid-lowering treatments on LDL-C in high and very high cardiovascular risk patients. The study is still being published, and only partial results are available.

Notably, despite having LDL-C levels above target values, 54.1% of patients received monotherapy, with statins being the most common (50.1%) followed by ezetimibe (1.8%) and PCSK9 inhibitors (1.7%). A total of 24% received combined therapy (16% with statin and ezetimibe).

### 1.4. Dual Therapy as First-Line Treatment

In light of the findings from these studies [12,13,14], it is clear that high-intensity statin monotherapy is often insufficient to achieve the targets recommended by current guidelines [1]. Various factors contribute to this, including adverse effects, patient education gaps, polymedication, drug availability and complexities in reimbursement for some of these treatments [16,17]. What is well demonstrated is that it is not the molecule that matters, but the common mechanism of these molecules to lower LDL cholesterol by inducing an increase in LDL receptors on the surface of hepatocytes [7,8,9]. Some experts advocate shifting the paradigm for very high-risk patients from “intensive first-line statin therapy” to “intensive lipid-lowering therapy”. A new approach for lipid-lowering therapy management, proposed in the European Heart Journal in 2021 [18] suggests initiation combined therapy based on the expected LDL-C reduction [18]. According to this approach [18], the first step would be a combination of statin and ezetimibe. If the target is not achieved (LDL-C < 55 mg/dL and reduction of more than 50%), a third lipid-lowering treatment should be added (bempedoic acid [19] or therapies targeting PCSK9 (monoclonal antibodies or siRNA)). Still, according to this algorithm, for extremely high-risk patients (post-acute coronary syndrome and history of vascular event within 2 years, post-acute coronary syndrome and presence of peripheral arterial disease/polyvascular disease, post-acute coronary syndrome and presence of multitruncal coronary disease or post-acute coronary syndrome and familial hypercholesterolemia), triple combination therapy could be initiated as first-line treatment.

In line with data from anti-hypertensive treatments [20], a single pill combining dual therapy is more effective than two separate components (two different pills) as the complexity of multi-drug regimens often leads to reduced patient compliance [20,21].

## 2. Materials and Methods

**Figure 1:**
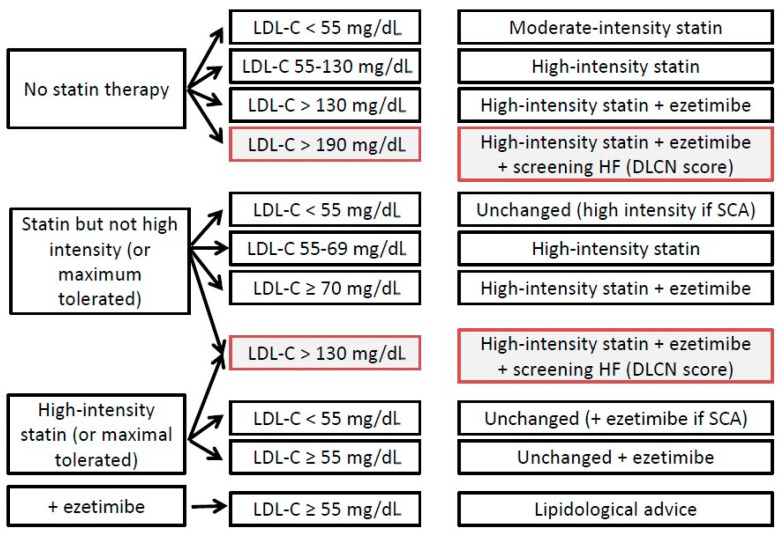
Algorithm for adapting lipid-lowering therapy in patients at very high cardiovascular risk. DLCN: Dutch Lipid Clinic Network; Ezetimibe: Ezetimibe 10 mg, FH: Familial Hypercholesterolemia; high-intensity statin: Atorvastatin (40 mg, 80 mg) or Rosuvastatin (20 mg, 40 mg); moderate-intensity statin: Atorvastatin (10 mg, 20 mg), Rosuvastatin 10 mg or Pravastatin 40 mg; ACS: Acute Coronary Syndrome. Red boxes: FH should be screened.

The aim of this single-center study is to assess the use of a pre-established lipid-lowering algorithm (Figure 1) specifically developed for our cohort, based on current clinical practices and guidelines, to achieve LDL-C targets in very high cardiovascular risk patients. This approach differs from the more recent algorithm proposed by Ray et al. (2022) [18] in the European Heart Journal, which advocates for combination lipid-lowering therapy as a first-line strategy in very high-risk patients. The proposed algorithm by Ray et al. [18] recommends starting with a combination of statins and ezetimibe for immediate and intensive lipid reduction, and further intensification with PCSK9 inhibitors or other agents if the targets are not met. Our study, however, evaluates a stepwise approach tailored to the local patient population and healthcare setting.

The research aimed to assess the number of patients reaching target levels after four to six weeks of treatment, as recommended by the guidelines and to determine, the number of patients still on treatment and their lipid profiles at one year.

Patients were assessed for cardiovascular risk, and a lipid profile was taken upon admission in the cardiology department. Cardiovascular risk was assessed using the SCORE system, which estimates the 10-year risk of fatal cardiovascular disease based on factors such as age, sex, smoking status, blood pressure, and cholesterol levels. Inclusion criteria were based on patients with documented atherosclerotic cardiovascular disease, diabetes with target organ damage, or severe chronic renal failure (eGFR < 30 mL/min/1.73 m^2^). Patients with known intolerance to statins or ezetimibe were excluded from the study. Patients at very high cardiovascular risk in primary and secondary prevention were screened for inclusion in the study.

Initially, the study included both primary and secondary prevention patients. However, due to the very small number of primary prevention patients (*n* = 6), which was insufficient for meaningful statistical analysis, we decided to focus exclusively on secondary prevention patients (n = 81). This decision was made to ensure a more robust and interpretable analysis of the effectiveness of the lipid-lowering algorithm in a more homogeneous patient population at very high cardiovascular risk.

As part of the routine practice, an algorithm for adapting lipid-lowering treatment (Figure 1) was implemented, based on the expected treatment efficacy (based on the literature’s data). Depending on the LDL-C level at admission and the treatment prior to admission, an adjustment was implemented at discharge.

Inclusion in the study was proposed to the patient at the end of hospitalization, with participation not affecting their management.

Patients were advised to follow a heart-healthy diet with reduced cholesterol intake, in accordance with ESC guidelines [1].

Inclusion lasted from July 2021 to March 2022. Anonymous data collection was conducted with the approval of the CHU UCL Namur ethics committee and the head of department.

Two telephone interviews were conducted to evaluate the persistence, adjustment, or discontinuation of lipid-lowering treatment:

- The initial contact occurred 4 to 6 weeks after the treatment initiation;
- The second contact took place, 1 year after the treatment initiation.

During this interview, any changes in the treatment regimen, as well as the reasons behind those changes, were documented.

In accordance with guidelines recommendations, lipid profiles were performed at 4 to 6 weeks and 1 year following treatment initiation. The results of these lipid profiles were collected via the patient’s medical records (local or regional) or the treating physician.

## 3. Statistical Analysis

Descriptive statistics, including means and standard deviations. Categorical variables were presented as frequencies and percentages

## 4. Results

### 4.1. Baseline Characteristics

A total of 81 patients were included, all in secondary prevention (Table 1). Most patients (62) were male, and the median age was 67 years (41 to 86 years). Common comorbidities included hypertension (59.3%), diabetes mellitus (23.5%), and chronic kidney disease (17.3%). Concurrent medications included ACE inhibitors (53.1%), beta-blockers (38.3%), and antiplatelet agents (53.1%).

A total of 46 (56.8%) patients were admitted electively and 35 (43.2%) via the emergency department.

The mean LDL-C level on admission was 117.2 ± 40.55 mg/dL and the median was 113 mg/dL.

A total of 49 (60.5%) participants had no lipid-lowering treatment (statin/ezetimibe/bempedoic acid/PCSK9 inhibitor) at inclusion. However, 18 (22.2%) were treated with a low- or moderate-intensity statin (low/moderate-intensity statins: Simvastatin (10 mg, 20 mg, 40 mg), Pravastatin (20 mg, 40 mg), Atorvastatin (10 mg, 20 mg), Rosuvastatin 10 mg), 11 (13.6%) with a high-intensity statin (high-intensity statins: Atorvastatin (40 mg, 80 mg), Rosuvastatin (20 mg, 40 mg)), 2 with combined therapy (statin and ezetimibe), and 1 with ezetimibe 10 mg alone.

### 4.2. Type of Treatment Initiated

Following the algorithm (Figure 1), high-intensity statin therapy was initiated in 30 (37%) patients, and combination therapy in 51 (63%) (Table 2).

### 4.3. Results at 4–6 Weeks

At 4–6 weeks post-hospitalization, we observed good adherence to the lipid-lowering treatment proposed by our algorithm, along with good tolerance (Table 3). A total of 69 (85.2%) patients adhered to the treatment on the telephone interview. Nonetheless, 6 (7.4%) discontinued the medication, and 6 (7.4%) were lost to follow-up.

Among the adherent participants (69), mean LDL-C level at 4–6 weeks was 54.3 ± 17.25 mg/dL. The LDL-C target (<55 mg/dL) was achieved in 36 of them (52.2%).

At this stage of follow-up, only one patient had his lipid-lowering treatment rapidly reduced (from rosuvastatin 20 mg to rosuvastatin 5 mg) due to poor general tolerance. However, no treatment intensification occurred at this stage.

### 4.4. Results at 1 Year

At 1-year follow-up (Table 3), 57 patients (70.4%) were adherent to their initial treatment, while 2 patients had their cholesterol-lowering treatment increased, 13 patients (16.1%) discontinued treatment, and 9 patients (11.1%) were lost to follow-up, withdrew from the study, or had died.

Among the adherent patients (57), the mean LDL-C at 1 year was 49.5 ± 16.9 mg/dL. The LDL-C target (<55 mg/dL) was achieved in 36 of them (63.2%).

Two patients had their cholesterol-lowering treatment increased at our center, due to not reaching the target at the intermediate lipid profile, despite good adherence to treatment. The first patient received inclisiran, and the second ezetimibe, in addition to their statin.

Of the adherent patients (57), 38 (66.6%) were treated with combination therapy and 19 (33.3%) with a high-intensity statin monotherapy. The mean LDL-C at 1 year for adherent patients on combination therapy was 50.42 ± 18.36 mg/dL with the LDL-C target achieved in 21 (55.3%). For the adherent patients on high-intensity statins, the mean LDL-C was 47.63 ± 13.74 mg/dL, and the target was achieved in 15 of them (78.9%).

### 4.5. Reasons Given for Discontinuing Treatment

Within the first month of treatment initiation, 6 patients (7.4%) discontinued their regimen, either spontaneously or on the advice of their attending physician. The reasons for discontinuation included poor compliance in two cases, poor general tolerance in two cases, myalgia in one case, and headache in one case.

At one year after treatment initiation, 13 patients (16.1%) discontinued treatment. Among them, six patients discontinued within the initial month, and seven patients discontinued between the second and twelfth months of treatment. Within the latter group, four patients stopped treatment without a specific rationale, one for liver enzyme disturbances, one reported diarrhea, and the last patient discontinued treatment due to CPK disturbances.

Table 4 summarizes the reasons for discontinuation of lipid-lowering treatment among the study participants during the follow-up period.

## 5. Discussion

The LDL-C target (<55 mg/dL) was successfully reached by 63.2% of patients at very high cardiovascular risk one year after initiation of their lipid-lowering treatment, which involved the early initiation of combination statin and ezetimibe therapy.

These results markedly outperformed those observed in the DA VINCI [12] study, where only 18% of very high cardiovascular risk patients (both primary and secondary prevention) achieved LDL-C targets across all treatment modalities. Moreover, only 22% of patients treated with high-intensity statins and 20% of patients receiving combination therapy achieved targets according to the 2019 guidelines on dyslipidemia [1].

The heterogeneity in results may be attributed to several factors. Firstly, the selection of statin intensity and the timing of combination therapy initiation likely contribute to this divergence. In the DA VINCI study [12], a majority (44.5%) of patients at very high cardiovascular risk received moderate-intensity statin monotherapy. Only 36.7% of patients were treated with a high-intensity statin and 9% with combination therapy of statin and ezetimibe. In contrast, our study introduced high-intensity statins in 37% of patients and combination therapy with statin and ezetimibe in 63%. No moderate-intensity statins were prescribed to our cohort.

This hypothesis was confirmed in the EUROASPIRE V [13] study, where 49.9% of patients at very high cardiovascular risk (secondary prevention) were treated with a high-intensity statin, and only 2.7% with combination therapy (statin and ezetimibe). After 4 to 12 weeks of treatment, less than 20% of patients achieved the previous targets (according to 2016 guidelines [15]), i.e., LDL-C < 70 mg/dL. The achievement of this LDL-C target value was 36.6% in patients on high-intensity statins. It is important to note that the EUROASPIRE V study was conducted when the 2016 ESC/EAS guidelines [15] were in effect, which set the LDL-C target for very high-risk patients at <70 mg/dL. These targets were subsequently updated in the 2019 guidelines [1], which further lowered the LDL-C target to <55 mg/dL to improve cardiovascular outcomes in very high-risk patients.

However, significant variability in results also exists among studies, even within patients treated with lipid-lowering therapies of similar intensity. While 63.2% of our patients achieved targets, only 22% and 20% of patients treated with high-intensity statins and combination therapy, respectively, did so in the DA VINCI study [12]. Similarly, in the EUROASPIRE V study [13], achieving the LDL-C target value (<70 mg/dL) was 36.6% in patients on high-intensity statins.

Several hypotheses may explain this discrepancy. Firstly, unlike the cited studies, our study protocol, patient inclusion, and follow-up were conducted in a lipid clinic, emphasizing patient empowerment [22,23].

Varied amounts of time were devoted to educating patients about dyslipidemia basics and potential side effects, treatment importance, and regular biological monitoring. Telephone contacts, in accordance with the protocol for data collection, were not only useful for collecting self-reported data but also served as an opportunity to reinforce the importance of adherence to therapy by reiterating key information. However, it is important to note that this method does not allow for an objective verification of patient compliance, as discussed in the biases.

Secondly, the smaller size of our cohort leads to less statistically robust results compared to larger cohorts in other studies.

Lastly, in other studies, the more intensive lipid-lowering treatments were reserved for a minority of patients. We could speculate that this minority corresponded to patients with very high LDL-C levels or had already received multiple lines of treatment. In this hypothesis, patients in our cohort would, for the same given treatment, have a lower mean/median LDL-C level, making the target more easily accessible.

This study has several limitations. First, the relatively small sample size (n = 81) may reduce the statistical power of the study, limiting the ability to detect smaller differences or generalize the findings to a broader population of very high-risk cardiovascular patients. Second, the use of telephone interviews to assess treatment adherence, while practical and helpful in reinforcing the importance of therapy, does not allow for objective verification of patient compliance. Self-reported data are inherently subject to recall bias and social desirability bias, which may affect the accuracy of the adherence rates reported. Despite these limitations, efforts were made to mitigate potential biases by using standardized questions and cross-referencing with available medical records whenever possible. Additionally, patients did not always give detailed reasons for discontinuation. Furthermore, our analysis focused solely on adherent patients at 4–6 weeks and one year. This bias sets us apart from other studies (DA VINCI [12], EUROASPIRE V [13]), where no distinction was made between adherent and non-adherent patients. It is important to note that our study started before bempedoic acid and inclisiran were available in Belgium.

## 6. Conclusions

In conclusion, based on the results of published clinical trials, and our population analysis, we suggest that the early initiation of combination therapy with statin and ezetimibe, or high-intensity statin, depending on the expected treatment efficacy, holds the potential to more rapidly and effectively achieve LDL-C targets in a larger proportion of very high-risk cardiovascular patients.

However, this necessitates active patient involvement, thorough treatment education, and regular monitoring, which could be facilitated in a lipid clinic.

## Figures and Tables

**Table 1:** Patient characteristics at inclusion.

Total Number	N = 81
Age—Years	
- Median	67
- Range	41–86
Male sex—Number (%)	62 (76.5)
Average LDL-C (mg/dL)	117.2 (±40.55)
Median LDL-C (mg/dL)	113
Usual treatment—Number (%)	
- No statin or ezetimibe	49 (60.5)
- Low/medium intensity statin	18 (22.2)
- High-intensity statin	11 (13.6)
- Dual statin/ezetimibe therapy	2
- Ezetimibe alone	1
- ACE inhibitors	43 (53.1)
- betablockers	31 (38.3)
- Antiplatelet agents	43 (53.1)
Elective patients—Number (%)	46 (56.8)
Patients admitted in emergency—Number (%)	35 (43.2)
Comorbidities—Number (%)	
- Arterial Hypertension	48 (59.3)
- Diabetes mellitus	19 (23.5)
- Chronic kidney disease	14 (17.3)

**Table 2:** Patient characteristics at inclusion.

Total Number	N = 81
High-intensity statin—Number (%)	30 (37)
Dual statin/ezetimibe therapy–Number (%)	51 (63)

**Table 3:** Endpoints at 4–6 weeks and at 1 year.

4–6 Weeks		1 Year
Continued treatment—Number (%)	69 (85.2)	57 (70.4)
- Average LDL-C (mg/dL)	- 54.3 ± 17.25	- 49.5 ± 16.9
- LDL-C target reached—Number (%)	- 36 (52.2)	- 36 (63.2)
Treatment stopped—Number (%)	6 (7.4)	13 (16.1)
Lost to view—Number (%)	6 (7.4)	9 (11.1)
Upgraded treatment—Number (%)	0	2

**Table 4:** Reasons for discontinuation of lipid-lowering treatment among study participants.

Reason for Discontinuation	Number of Patients	Percentage (%)
Poor compliance	2	2.5
Poor general tolerance	2	2.5
Myalgia	1	1.2
Headache	1	1.2
Elevated liver enzymes	1	1.2
Diarrhea	1	1.2
CPK disturbances	1	1.2
Discontinuation without specific reason	4	4.9
Total	13	16

## Data Availability

The original contributions presented in the study are included in the article, further inquiries can be directed to the corresponding author.
